# Posttranscriptional 3′-Terminal Modifications of *Escherichia coli* RNA Fragments Evolved for Diversity Boosting

**DOI:** 10.3390/microorganisms13092189

**Published:** 2025-09-19

**Authors:** Nikita M. Kamoldinov, Valery V. Panyukov, Nikolay P. Kolzhetsov, Natalia Y. Markelova, Konstantin S. Shavkunov, Uliana S. Shvyreva, Olga V. Alikina, Olga A. Glazunova, Iuliia A. Praslova, Olga N. Ozoline

**Affiliations:** 1 Department of Functional Genomics of Prokaryotes, Institute of Cell Biophysics of the Russian Academy of Sciences in Federal Research Center Pushchino Scientific Center for Biological Research of the Russian Academy of Sciences, 142290 Pushchino, Russia; nmk44@mail.ru (N.M.K.); panyukov@itaec.ru (V.V.P.); kolya.kolzhecov@mail.ru (N.P.K.); markelova.n.y@gmail.com (N.Y.M.); shavkunovks@gmail.com (K.S.S.); uliana.shvyreva@gmail.com (U.S.S.); alikina.olga@mail.ru (O.V.A.); glazunova.olga.a@gmail.com (O.A.G.); juliapraslova2828@mail.ru (I.A.P.); 2 Faculty of Biotechnology, Lomonosov Moscow State University, 119991 Moscow, Russia; 3 Department of Bioinformatics, Institute of Mathematical Problems of Biology RAS—The Branch of Keldysh Institute of Applied Mathematics of the Russian Academy of Sciences, 142290 Pushchino, Russia

**Keywords:** posttranscriptional RNA modifications, 3′-end extensions, polynucleotidilation, chimeric oligonucleotides, leucine tRNAs, Dps, secreted RNAs

## Abstract

An emerging area of microbial biology focuses on oligonucleotides excised from functional RNAs and subsequently fulfilling an independent cellular role. Some of these products are subjected to modifications that may expand their functional inventory. Here, we applied a differential analysis of intra- and extracellular RNA fragments produced by wild-type *Escherichia coli* and its *dps*-null mutant and discovered leucine tRNA fragments with random 3′-terminal extensions among oligonucleotides with Dps-dependent secretion. We observed an exclusive intracellular enrichment of modified LeuT(VPQ) tRNA fragments compared to secretomes, with abundance level dependent on growth medium and the presence of competing bacteria. To assess the pervasiveness of this phenomenon, we developed a custom computational pipeline for detecting variable RNA termini in RNA-seq data. Beyond LeuT(VPQ) tRNA fragments, several other genomic loci yielded oligos with highly heterogeneous ends, indicating that terminal elongation, most prevalent in LeuT(VPQ), is not exclusive to these fragments. Ex vivo testing using synthetic LeuT(VPQ) analogs revealed their stimulatory effect on the persistence of multiple taxa in an artificial microbiome, which was attenuated by 3′-end elongation. We propose that non-template extensions may serve to broaden the spectrum of target molecules for elimination of unused mRNAs by an interference-like mechanism or to generate sequences absent from the *E. coli* genome as part of a primitive defense system.

## 1. Introduction

Being found in all classes of RNAs, posttranscriptional modifications may be considered as functional tools in various processes in living cells [1,2]. The possible effects from these modifications can not only result in altered structure and stability of transcripts and their fragments [3] but also influence interaction with proteins [4,5] and affect gene expression [6]. The structural impact is presumed to be the major outcome from RNA modifications [5]. By affecting the intrinsic physicochemical properties of oligonucleotides, they can be important players in macromolecular interactions [7].

More than 170 posttranscriptional alterations have been registered to date [8] across all domains of life [9], many of which are well-studied and have a clear biological role. A wide range of chemical modifications has been described as functionally relevant elements of transfer and ribosomal RNAs. For instance, pseudouridine (Ψ) protects tRNA and rRNA molecules from cleavage, stabilizes their regional structures, and prepares rRNAs for proper ribosome assembly, decoding of mRNAs, and translation [10]. Inosine in tRNAs plays an essential role in wobble base pairing during translation [11]. Being a prevalent type of RNA modification in eukaryotes [12], N6-methyladenosine regulates numerous cellular processes, including differentiation [13], splicing [14], mRNA translation [15], and stability [16]. In bacteria, the role of m6A is apparently not so well-studied, and though some authors suggest functional significance of this abundant nucleotide [17], recent studies raise doubt on its high relevance for bacterial cells and suggest an absence of a direct enzymatic recognition mechanism for its formation [18].

A particular group of modifications includes terminal extensions of RNA molecules, and the addition of adenines to the 3′-ends of messenger RNAs is known to exist both in higher organisms and bacteria [19,20]. However, unlike in eukaryotes, where polyadenylation ensures mRNA stability [21], in *E. coli* and other bacteria, it is rather used as a decay signal: poly(A) polymerase I (PAP I) adds poly(A) tails to transcripts, marking them for degradation and assisting in RNA quality control [22].

Another enzyme, PNPase, is bifunctional and can both degrade RNAs via 3′→5′ phosphorolysis and, under certain conditions, add heteropolymeric (A-rich) non-template extensions at their 3′-ends, especially in products originated from Rho-dependent transcription terminators [22,23,24]. Due to this ambiguity, debates are ongoing on its biological significance: whether to consider the PNPase tagging function as a separate process that provides accessible single-stranded overhangs for degradosome-mediated decay [25] or nucleotides are added as a by-product during stalled degradation caused by secondary structures or pauses [26]. According to this concept, structural or sequence barriers may lead to cycles of addition or degradation of a few nucleotides, so the observed tails are an intrinsic outcome of phosphorolysis/polymerization balance rather than a targeted tailing action [22]. Only part of transcripts undergo polyadenylation in bacteria, and in *E. coli*, substrate selection is presumed to be determined by the low level of PAP I, as witnessed from a significant increase in the number of polyadenylated transcripts associated with induced overproduction of this enzyme [27,28]. In vivo, RNA-binding factors, including Hfq, CspE, and the S1 protein of the ribosome, may influence whether PNPase acts predominantly as polymerase or exonuclease [22]. Notably, PAP I can also interact with Hfq, as well as with PNPase and RNase E in vivo and in vitro [29,30], and the interplay between these enzymes has been proposed to play a major role in deciding between PAP I and PNPase as the polymerizing enzyme [22].

Thus, a hallmark of contemporary molecular biology is the increasing focus on the regulatory roles of short RNAs (≤40 nt) derived from processed transcripts of functional genes. Through structural rearrangements or processing-induced modifications, some of these molecules may develop independent cellular or extracellular functions. Assuming the potential regulatory significance of short RNAs with secondary biogenesis in intercellular communications, we have initiated a comprehensive investigation of bacterial RNA secretomes. These extracellular molecular assembles form composition profiles markedly distinct from intracellular transcriptomes [31,32,33,34], suggesting potential signaling and/or regulatory functions. However, such activities would require both the selective export of functional oligonucleotides and their protection from degradation. Posttranscriptional modifications may serve as markers of RNAs designed for secretion and favor their stability maintenance. To investigate this paradigm, we conducted a targeted search for regulatory RNA fragments in *E. coli* through comparative analysis of intracellular and extracellular RNA fractions from the wild-type K-12 MG1655 and its Δ*dps* mutant strains. We selected the nucleoid protein Dps as a candidate transport factor based on its ability to bind both DNA and RNA [35,36,37,38,39,40] along with several reports of its association with membrane structures [41,42]. Comparative analysis revealed over one hundred transcribed genomic loci with reduced abundance of products in the Δ*dps* secretome, maintaining wild-type or higher levels inside mutant cells [43]. Among specifically secreted oligonucleotides, we identified a nanomere (5′-GCCAAGGCG-3′) frequently attaching to fragments of processed RNAs, i.e., participating in the rarest posttranscriptional modification—chimerization. At frequencies exceeding random ligation probabilities, it formed chimeric molecules predominantly derived from tRNAs. In this study, we expanded the previously undertaken differential analysis to focus on the most frequent posttranscriptional modification—non-template nucleotide incorporation at the 3′-ends of processed RNAs. We revealed striking diversity in the 3′-ends of oligonucleotides derived from five of eight leucine tRNAs, proved exclusive non-random extension patterns in fragments processed from four leucine tRNAs (LeuT, LeuV, LeuP, and LeuQ), and witnessed functional implications of random 3–9 nucleotide extensions incorporated at their 3′-termini.

## 2. Materials and Methods

### 2.1. Bacterial Strains

We used *Escherichia coli* K-12 MG1655 wild-type strain and its *dps*-null mutant (Δ*dps*), constructed as described in [44], to identify RNAs with Dps-dependent secretion and to assess their influence on bacterial growth in monoculture. For Dps overproduction, we used *E. coli* BL21(DE3) cells, and *Rhodospirillum rubrum* ATCC 11170 (*R. rubrum*) was implemented as an external control taxon in artificial microbiome experiments.

### 2.2. Extracellular Dps Detection (Western Blot Analysis)

*E. coli* strains K-12 MG1655, Δ*dps* mutant, and BL21(DE3) were cultured overnight in 25 mL of M9 medium. Cells from 1 mL aliquots were precipitated, resuspended in 100 µL of loading buffer (0.125 M Tris-HCI (pH 6.8), 4% SDS, 20% glycerol, 0.002% bromphenol blue, 10% β-mercaptoethanol), and lysed by heating at 90 °C for 10 min. Subsequently, 15 µL samples were analyzed by electrophoresis in 12.5% SDS-PAGE. For extracellular protein analysis, 20 mL of spent medium from *E. coli* K-12 MG1655 and BL21(DE3) cultures was collected after cell removal and concentrated to 200–360 µL using Amicon^®^ Ultra 3K centrifugal filters (Millipore, Burlington, MA, USA). Following electrophoretic fractionation, proteins were transferred to a PVDF membrane. For Dps detection, the membrane was treated with rabbit anti-Dps antibodies [44] followed by horseradish peroxidase-conjugated goat anti-rabbit IgG secondary antibodies. Protein bands were visualized using Pierce™ ECL Western Blotting Substrate (Thermo Scientific, Waltham, MA, USA) and imaged on an iBright™ CL750 Imaging System (Thermo Scientific, Singapore). Band intensities were quantified using ImageJ software (v1.52) [45]. For this experiment, recombinant Dps was purified from *E. coli* BL21(DE3) cells transformed with the pGEMΔXba-*dps* plasmid, constructed as described in [46]. Transformed cells were cultured in LB medium supplemented with ampicillin (100 μg/mL) at 37 °C. Gene expression was induced by 0.02 mM IPTG at mid-log phase (OD600 0.4–0.6), followed by 12 h of incubation for Dps production. The purification protocol consisted of sequential ion-exchange chromatography (DEAE-Sephadex A-25, GE Healthcare, Uppsala, Sweden) and gel filtration using Sephadex G-200 (Pharmacia/Cytiva, Uppsala, Sweden). The protein was then concentrated using Vivaspin-20 centrifugal concentrators (Sartorius, Goettingen, Germany), dialyzed against storage buffer (50 mM Tris-HCl (pH 7.5), 50 mM NaCl, 0.1 mM EDTA, 5% glycerol), and stored in the freezer.

### 2.3. RNA Purification and Sequencing

Intracellular RNAs were isolated from 15 mL of bacterial cultures using TRIzol reagent (Invitrogen, Carlsbad, CA, USA) and following protocols optimized previously [32,33,47]. Small RNA enrichment was performed using the mirVana miRNA Isolation Kit (Ambion, Austin, TX, USA). For extracellular RNA (exoRNAs) isolation, 1 mL of cell-free culture medium was obtained by centrifugation (2000 rpm, 20 min) followed by double filtration through 0.22 μm sterile syringe filters. exoRNAs were purified using the miRNeasy Serum/Plasma Kit (Qiagen, Hilden, Germany), and their concentrations were estimated using Qubit 3.0 fluorometer (Thermo Fisher Scientific, Singapore) and Qubit RNA HS Assay Kit (Thermo Fisher Scientific, Eugene, OR, USA). RNA sequencing libraries were prepared according to the standard protocol of Ion Total RNA-Seq Kit v2 (Thermo Fisher Scientific, Carlsbad, OR, USA) with minor modifications. For precise identification of oligonucleotide termini, RNA samples underwent 5′- and 3′-ends adapter ligation. Following reverse transcription, samples were purified using AMPure XP (Beckman Coulter, Brea, CA, USA). PCR amplification was performed with 0.7 μL of each v2 primer, after which the products were purified with the Monarch PCR & DNA Cleanup Kit (New England Biolabs, Ipswich, MA, USA). Size selection was carried out by electrophoresis in a 6% polyacrylamide gel using Quick-Load pBR322 DNA-MspI Digest DNA Ladder (New England Biolabs, Ipswich, MA, USA) as a length marker. Gel areas with cDNA amplicons (90–123 bp) were excised and minced. Target products were eluted overnight using an elution buffer from the NEBNext Multiplex Small RNA Library Prep Set for Illumina (Set 1) (New England Biolabs, Ipswich, MA, USA). Subsequent sample preparation steps, including cDNA precipitation, pellet washing, and resuspension in 10-fold diluted TE buffer, were performed following the NEBNext Multiplex Small RNA Library Prep Set for Illumina (Set 1) protocol. Library concentration was estimated using Qubit 3. Libraries were diluted to 100 pM and subjected to emulsion PCR using the Ion OneTouch^TM^ 2 System with Ion PGM Hi-Q View OT2 Kit (Thermo Fisher Scientific, Carlsbad, CA, USA). Sequencing was performed on an Ion Torrent PGM analyzer (Thermo Fisher Scientific, Carlsbad, CA, USA), and the resulting sequencing libraries were deposited in the NCBI SRA database (Table 1).

### 2.4. Pre-Processing of RNA-seq Datasets and Their Mapping to the Genome

Quality filtering was performed using the filter by quality tool on the UseGalaxy.org platform [48], retaining only reads with ≥90% of nucleotides meeting a Q20 quality threshold. Adapter sequences were removed by Ion Torrent PGM software (version 5.14), followed by an additional trimming of 3′-end sequences containing ATCACCGACTGCCCA(N)_n_ (the 5′-terminal sequence of adapter) motifs. Trimmed reads were sorted by length (16–50 nt), and fragments of each size were separately aligned to the genome of *E. coli* K-12 MG1655 (NCBI RefSeq accession NC_000913.3) [49]. For 5′-end mapping of the obtained reads, we used Matcher software (http://www.mathcell.ru/DnaRnaTools/Matcher.zip, accessed on 28 July 2025) [32,44,50], requiring precise coincidence with the genome. Reads with sequences matching several genomic loci were attributed to all of them in equal proportions. Reads with different lengths in each position were summarized, providing the profile of their 5′-ends distribution over the entire genome.

### 2.5. Search for Oligonucleotides with Dps-Dependent Secretion

The search for genomic regions producing potential signaling exoRNAs was carried out as described in [43] with modifications aimed to increase the sensitivity of the approach for detecting modified oligonucleotides. Matcher-derived profiles were normalized per 1 million quality-controlled reads (16–50 nt) in each of the eight experiments (Table 1). For differential analysis, transcript counts were summed within sliding 5 bp windows (1 bp step size). The mean read counts across four types of paired experiments for each window were then calculated. For genomic loci, yielding at least 25 reads, the ratio of product abundances among the wild-type and mutant strains was calculated. Candidate selection required the following: (1) ≥1.5-fold reduced abundance in the Δ*dps* mutant secretome and (2) comparable or elevated levels in the Δ*dps* intracellular transcriptome. For each candidate region, we identified the peak position with maximum read number and compared the wild-type and mutant transcriptional outputs.

### 2.6. Search for Textual Deviations from the Genomic Sequences

**Figure 1:**
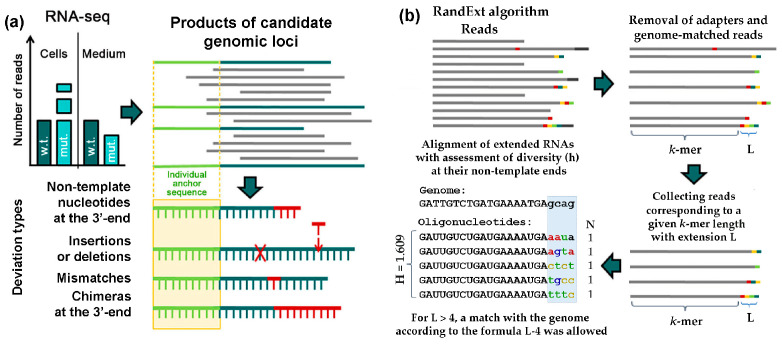
Schemes illustrating (**a**) the strategy used for the selection of RNAs with presumably Dps-dependent secretion and (**b**) for identifying RNA fragments with random extensions (RandExt).

Reads derived from the selected genomic loci were assembled using unique “anchors” defined by 5′-ends corresponding to peak maxima and sufficient length (12–14 nt) to ensure specificity (Figure 1a).

The obtained reads were aligned to the *E. coli* K-12 MG1655 genome using either Excel sorting option or MUSCLE [51] available at the EMBL-EBI service platform. The identified sequence deviations were classified into four categories: (1) Non-template additions: 1–3 nt extensions of any sequence at either end or longer extensions containing only 1–2 distinct nucleotides. (2) Chimeras: ≥4 nt extensions at any end containing ≥3 distinct nucleotides. (3) Insertions or deletions (indels) and (4) base substitutions (mismatches) occurring outside terminal triplets. A total number of reads with such modifications was required to accurately quantify the percentage of reads containing 3′-end extensions, which were the focus of this study. For classification purposes, each read was counted as a single erroneous sequence even when containing multiple deviation types, though all individual deviations were recorded for frequency calculations. Deviation frequencies were calculated as mean values from two experimental replicates when each contained ≥100 reads. For sample sets containing fewer reads, the outputs of two replicate experiments were either pooled or ignored (if the total count was less than 80).

### 2.7. Search for Oligonucleotides Containing Random Sequences at the 3′-End (RandExt Algorithm)

The RandExt algorithm has been developed to detect transcripts containing random sequences at the 3′-ends (Figure 1b). The software processes oligonucleotides of user-defined length determined by both the target *k*-mer size at the 5′-ends and the desired length (L) of non-templated 3′-sequences. At the preliminary stage, the algorithm identifies and removes the remnants of the 3′-adapter by its 5′-terminal sequence (ATCACCGACTGCCCA) in the reads and also filters out reads that perfectly match the genome. Since our Ion Torrent PGM sequencing datasets contained negligible 5′-adapter contamination (if any), the algorithm does not include 5′-adapter removal option. The remaining reads are aligned to the genome, and those that match the genome sequence exceeding the specified *k*-mer length are ignored. For reads containing sequence differences from the genome, copy numbers are determined, and sequence diversity is then assessed using the Shannon index: H = −Σp_i_ × Ln(p_i_), where p_i_ represents the frequency of each unique oligonucleotide. For flank analysis with L = 4, we required complete sequence divergence from the genome. For longer extensions, L-4 matching nucleotides were permitted. The algorithm is available at: https://mathcell.ru/prog/StudyRna.rar (last accessed on 25 July 2025).

### 2.8. Datasets Used to Analyze LeuTVPQ Extensions Depending on Culture Media, the Presence of Competing Bacteria, and Exogenous Oligonucleotides

To examine the influence of growth conditions on LeuTVPQ terminal modifications, we analyzed sixteen RNA-seq datasets previously obtained from *E. coli* intracellular transcriptomes or secretomes [32,33,43,52]. All datasets contained fraction of short oligonucleotides, from cells grown in either M9 medium or Luria–Bertani (LB) broth (The six key datasets are listed in Table 2). The datasets were processed and evaluated as described in Section 2.4, except that LB-born RNAs were removed from the datasets, as described in [33].

### 2.9. Evaluation of the Effect of LeuTVPQ with Non-Template Nucleotides at the 3′-Ends on the E. coli Growth in Monoculture

Growth experiments were carried out in 0.2 mL volume in 96-well TC plates (suspension, F (Sarstedt AG & Co, Nümbrecht, Germany) in Synergy H1 Hybrid Multi-Mode Reader (BioTek Instruments Inc., Berlin, Germany)) at 37 °C with agitation. Depending on the task, M9 mineral medium, Luria–Bertani broth (tryptone 1.0 g/L, yeast extract 0.5 g/L, and NaCl 1.0 g/L), or complex Gut Microbiome Medium (GMM) was used in these experiments. GMM contained tryptone peptone, 0.2%; yeast extract, 0.5%; D-glucose, 2.2 mM; L-cysteine, 3.2 mM; cellobiose, 2.9 mM; maltose, 2.8 mM; fructose, 2.2 mM; meat extract, 0.5%; KH_2_PO_4_, 10 mM; MgSO_4_ × 7H_2_O, 0.008 mM; NaHCO_3_, 4.8 mM; NaCl, 1.37 mM; Tween-80, 0.05%; CaCl_2_, 0.072 mM; resazurin, 4 μM; vitamin K, 5.8 μM; FeSO_4_ × 7H_2_O, 1.44 μM; histidine–hematin stock solution, 0.1% (1.2 mg/mL hematin in 0.2 M histidine); vitamin mix, 1.0%; and trace mineral mix, 1.0%, pH 7.0. Filter-sterilized vitamin K, FeSO_4_ × 7H_2_O, histidine–hematin solution, vitamin mixture, and trace element mixture were added to the total volume of autoclaved, sterilized medium before inoculation. An inoculum of *E. coli* K12-MG1655 was prepared by diluting an overnight culture to OD_600_ = 0.5. This inoculum was then added to fresh medium at a 1:500 dilution, yielding approximately 10^5^ CFU/mL. For the assay, 190 μL aliquots of inoculated culture were dispensed into plate reader wells, followed by addition of 10 μL of either dissolved synthetic RNAs (Syntol, Moscow, Russia) or sterile water as control. Synthetic analogs of oligonucleotides (Table 3) were added to the bacterial culture to a final concentration ranging from 25 nM to 5 μM, and the bacterial growth was carried out at 37 °C under aerobic conditions with dynamic monitoring by measuring OD_600_ for 24–48 h.

To assess the functionality of terminal extensions, three synthetic analogs of model oligonucleotides containing random sequences at the 3′-end (LeuTVPQ_3N-9N) were used (Table 3). Their effect was compared with the influence of oligonucleotides with genomic sequences of the same length in growth experiments using LB and GMM media.

### 2.10. Revival of Rat Fecal Bacteria in GMM and Their Treatment with Synthetic Oligonucleotides

The medium prepared as described above was heated at 80 °C to ensure complete dissolution of all components, then cooled under CO_2_ flow and transferred to N_2_-purged Hungate tubes. After sealing, the tubes were sterilized at 121 °C for 20 min. Fecal material was collected from SPF-grade male Wistar rats aged 10–12 weeks and weighing 250–280 g. The animals were kept in nursery for laboratory animals, “Pushchino”, on standard rodent chow with ad libitum tap water. Fecal samples (0.2–0.3 g) from five laboratory rats were homogenized in 1 mL of GMM, as suggested in [53]. Following homogenization, insoluble particles were pelleted using a spinner, and the supernatant from each of homogenized fecal samples was aseptically transferred into Hungate tubes containing 10 mL of fresh GMM using sterile syringes. The inoculated medium was cultured at 37 °C for 48 h with gentle agitation. The resulting culture was then divided equally into five aliquots and transferred to fresh Hungate tubes containing an equivalent volume of GMM. One aliquot from each rat fecal sample was used as a control. The remaining four aliquots were supplemented with the model oligonucleotides listed in Table 3. Oligonucleotides T6 and 6N were added to a final concentration of 200 nM, while T9 and 9N were added to 400 nM. These concentrations were selected based on their previously observed ability to affect the growth of *E. coli* in monoculture and the availability of the synthetic oligonucleotides. The cultures were maintained at 37 °C, with the second addition of oligos at identical concentrations after 24 h, followed by continued cultivation for another 24 h.

Bacterial cultures from all twenty-five samples were pelleted by centrifugation (3000 rpm, 20 min) and washed twice with sterile PBS. Genomic DNA was then extracted using the EasyPure^®^ Stool Genomic DNA Kit (TransGen, Beijing, China), following the manufacturer’s protocol for pathogenic bacteria. DNA was eluted from the filters by applying two sequential 30 μL aliquots of elution buffer. DNA concentration was measured using a Qubit 3.0 fluorometer (Thermo Fisher Scientific, Singapore), while sample purity was assessed by measuring A_260_/A_280_ and A_260_/A_230_ ratios on a NanoDrop ND-1000 spectrophotometer (Thermo Scientific, Wilmington, DE, USA).

### 2.11. Amplicon Sequencing of 16S rRNA Genes for Taxonomic Analysis

The impact of model oligonucleotides on ex vivo cultured fecal bacteria was evaluated by 16S rRNA phylotyping. PCR amplification was carried out in 2 × 25 μL reactions using Veriti 96-well Thermal Cycler (Applied Biosystems, Thermo Fisher Scientific, Foster City, CA, USA) with Q5 High-Fidelity DNA Polymerase (New England BioLabs, Ipswich, MA, USA) and universal primers 27f and 1492r (Evrogen, Moscow, Russia). Prior to amplification, DNA samples were normalized to 50 ng/μL, and 1 μL per 25 μL reaction was used as a template. The thermal cycling protocol consisted of initial denaturation (95 °C for 5 min), 30 cycles of synthesis (95 °C for 1 min, 59 °C for 1 min, 72 °C for 1.5 min), and a final extension (72 °C for 5 min). Amplification efficiency was verified by electrophoresis of 2 μL aliquots of PCR products in a 1% agarose gel with ethidium bromide staining. Amplicons were then purified using AMPure XP magnetic beads (Beckman Coulter, Brea, CA, USA). Sequencing libraries were prepared using the Native Barcoding Kit 96 V14 (SQK-NBD114.96) protocol, with modified incubation times for enzymatic reactions: the DNA repair step using NEBNext^®^ Ultra™ II End Repair/dA-Tailing Module (New England Biolabs, Ipswich, MA, USA) was extended to 25 min, and the ligation step was run for 60 min. The barcoded libraries were then loaded onto a PromethION Flow Cell (R10.4.1) and sequenced. The sequencing parameters selected in the MinKNOW GPU v23.04.3 program were quality threshold—QC 9; basecalling model—high accuracy (Dorado 7.2.11 algorithm, dna_r10.4.1_e8.2_400bps_hac@v4.3.0); and activated demultiplexing function with removal of barcodes and adapters. Complete sequencing statistics for all samples are provided in Supplementary Table S1.

### 2.12. Taxonomic Analysis of Ex Vivo Cultured Bacterial Communities

The obtained amplicons were taxonomically classified using EPI2ME Desktop V5.2.2 software within the metagenomics workflow based on Kraken2 classifier [54]. The resulting datasets contained 203,684–414,638 sequences successfully classified for 195 bacterial genera. Relative taxon abundances were calculated as percentages of total reads in each of the 25 datasets. To enable these calculations, we added one pseudocount to genera that received zero assigned reads, thereby preventing division by zero errors. To minimize inconsistency from low-abundance genera, we applied the following inclusion criteria: for taxa showing increased abundance, the experimental sample required ≥0.01% representation, and for suppressed taxa, the control sample required ≥0.01% presence. Genera meeting these thresholds in at least four of five paired samples were retained for downstream analysis. For each qualifying genus, we calculated fold change ratios (FCRs) and averaged them across replicates (n = 4–5). Standard deviations (StDs) and Z-scores were computed in Excel, with FCR = 1 as the null hypothesis. For Z-scores < 8, *p*-values were calculated using Excel 1-NORM.S.DIST(Z,TRUE) function. Z-scores ≥ 8 were analyzed separately in R to ensure accurate extreme value estimation.

### 2.13. Statistics

Statistical analyses were performed using SigmaPlot (v11.0). We first assessed data normality using the Shapiro–Wilk test. If the datasets violated normality assumptions, group comparisons were conducted using the non-parametric Mann–Whitney U test (via SigmaPlot “Compare Two Groups” function) to evaluate median differences. To evaluate the statistical significance of posttranscriptional modifications, we combined replicates from wild-type *E. coli* K-12 MG1655 and Δ*dps* mutant strains. *p*-values were calculated across four datasets, with Bonferroni correction applied for multiple comparisons. Due to non-normality, variability was estimated using mean absolute deviations (MAD) instead of two-way ANOVA. We used the Pearson correlation coefficient (R) to assess the consistency of RNA-seq datasets. The statistical significance of R was estimated using a VassarStats online tool (http://vassarstats.net/tabs_r.html, last accessed on 25 July 2025) [55].

## 3. Results

### 3.1. Dps Protein Found in Culture Milieu May Promote the Release of RNAs from Cells

**Figure 2:**
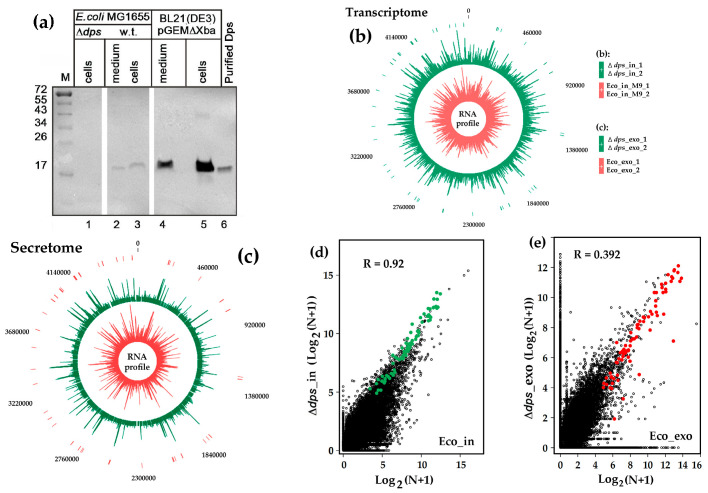
Western blot hybridization (**a**) revealed the presence of Dps outside *E. coli* cells, while differential RNA-seq analysis (**b**,**c**) identified short RNAs with potential Dps-dependent excretion. (**a**) Cellular samples (lanes 1, 3, and 5) were 10-fold concentrated. Extracellular fractions were concentrated 50- and 27.8-fold for *E. coli* MG1655 and BL21(DE3) samples, respectively. (**b**,**c**) Profiles show the normalized distribution of 16–50 nt reads isolated from bacterial cells (**b**) or secretomes (**c**) and plotted as Log_2_(N+1). (**d**,**e**) Correlation plots for the number (N) of the same reads obtained in the indicated RNA-seq experiments. Colored symbols correspond to RNAs whose intracellular content of the mutant strain remained at the baseline or increased (**d**) while decreasing in its secretomes (**e**). Corresponding positions in the genomes are marked by ticks (**b**,**c**).

Inspired by literature data on the presence of nucleoid protein Dps in membrane structures [41,42] and keeping in mind our pull-down assay results indicating Dps’ ability to retain short RNAs in vitro [39], we performed Western blotting to rate the amount of Dps present in the culture medium (Figure 2a). As expected, no reactivity with anti-Dps antibodies was found in the *E. coli*_Δ*dps* mutant lysates (Figure 2a, lane 1), whereas *E. coli* K-12 MG1655 cell population accumulated a detectable amount of this protein both inside and outside the cells (lanes 2 and 3). Induction of the recombinant *dps* expression from the pGEMΔXba-*dps* vector in *E. coli* BL21(DE3) cells gave an obvious increase in the amount of this protein (lanes 4 and 5). Quantitative analysis using ImageJ showed that 0.34 ± 0.07% of total Dps is located extracellularly in the wild-type strain, and in *E. coli* BL21(DE3) culture, its extracellular level was approximately at the same level (0.52 ± 0.12%). Thus, although Western blotting has inherent limitations as a precise quantitation method, and the sensitivity of two strains to excess Dps may be different, it is likely that overproduced Dps does not reach cytotoxic concentrations requiring its active export and adaptation at the level of gene expression. We assumed that Dps’ appearance in culture medium, either through membrane translocation or due to release from lysed cells, could enhance extracellular accumulation of short RNAs. To test this assumption, we conducted differential RNA-seq analysis comparing the secretomes and transcriptomes of the wild-type and Δ*dps* mutant strains. Eight RNA-seq experiments were performed. Four of them (denoted by asterisks in Table 1) were carried out simultaneously, with parallel isolation of intracellular and secreted RNAs from both strains. Four other experiments were conducted separately to assess the reproducibility of transcriptomic data.

The obtained sequence reads were aligned to the *E. coli* MG1655 genome, using the option of Matcher software [50,55] when only reads ideally coinciding with the genomic sequence were retained, and only their 5′-ends were mapped. Normalized expression profiles demonstrated a strong correlation between replicates, which was maximal for two samples with intracellular RNA fragments from the Δdps mutant (R = 0.98). An inferior but still high correlation (R = 0.58) was observed for two extracellular RNA sets (exoRNAs) from the mutant strain. Two wild-type replicates correlated with R = 0.90 (intracellular samples Eco_in_M9_ 1 and 2) and R = 0.65 (extracellular samples Eco_exo 1 and 2).

Figure 2b,c display four profiles averaged over two paired experiments. The clear difference between the internal profiles in Figure 2b,c confirms an already known difference in the spectra of intracellular and secreted RNAs, respectively [31,32,33]. The difference between the outer and inner profiles in panel 2b reflects variations in the transcriptional output in the parental strain and the Δ*dps* mutant. Although the ability of Dps to perform a regulatory role in gene expression remains greatly uncharacterized [56], its non-random distribution across the *E. coli* genome [44] may contribute to this difference by making DNA accessible or inaccessible to transcriptional machinery. However, the absence of Dps appears to have only moderate effects on the intracellular transcriptome, as evidenced by the strong correlation (R = 0.92) between the wild-type and mutant RNA-seq profiles (Figure 2d). In contrast, secretome profiles showed minimal similarity (R = 0.392, Figure 2e), indicating their pronounced Dps dependence. This dramatic loss of correlations resulted from numerous oligonucleotides with a high difference in the abundance patterns. As shown in Figure 2e, many data points align along the axes, representing oligonucleotides that are highly abundant in the Eco_exo samples while being at almost the background level in the mutant secretomes (or vice versa). Thus, the RNA secretome is clearly sensitive to Dps.

### 3.2. RNA-Seq Analysis Revealed Oligonucleotides with Potential Dps-Dependent Secretion

Following our previous strategy [43], we combined replicate profiles to identify genomic regions, yielding products with ≥1.5-fold decreased representation in the Δ*dps* mutant secretomes, maintaining wild-type or higher levels in intracellular transcriptomes. The previous RNA-seq analysis revealed 132 genomic loci meeting these criteria [43]. Fragments of tRNAs and sRNAs, the genes of which occupy only 0.14% and 0.4% in the genome, were significantly overrepresented among these regions, accounting for 23.5% and 19.7% of the identified loci, respectively. A similar binding preference was observed in our pull-down experiments with Dps immobilized on acrylate beads and short intracellular RNAs from *E. coli* [39]. Compared to unmodified acrylate bead control, we identified 42 oligonucleotides with at least 5-fold higher abundance in eluates from Dps-modified beads. Of these, 24 originated from 12 distinct tRNAs and 6 from various sRNAs. Therefore, structured tRNA and sRNA fragments have a preferential affinity to Dps. The rationale to reevaluate the previous RNA-seq analysis was based on two key observations: fragments of only 10 types of tRNAs were identified as potential Dps partners, and not all homologous tRNAs exhibited similar dependence on Dps [43]. To understand the biological basis of this apparent selectivity, we developed an improved search strategy for the current study (Table 4).

By changing the normalization of datasets from the number of genome-mapped reads to the number of quality-controlled reads, we accounted for the contribution of unmapped reads, the moiety of which contains modified oligonucleotides. By increasing the minimum length of analyzed reads from 12 to 16 nucleotides, we excluded predominantly unmodified short oligonucleotides. Finally, by reducing the running window from 10 to 5 bp and adding a peak pinpointing step, we ignored oligonucleotides differing in 5′-ends. These refinements still yielded 102 genomic loci meeting our criteria for differential transcriptional output and secretion between the wild-type and mutant strains (Supplementary Table S2). This final set contains one or even two fragments derived from different stem-loop structures of 19 tRNA types, including SelC. However, fragments of all isoleucine and methionine tRNAs, as well as oligonucleotides derived from homologs of some selected tRNAs, were not identified. Their inspection in most cases revealed only quantitative deviation from the criteria used (Table 4). However, fragments derived from methionine and arginine tRNAs, along with *ileV* and *valT*(*Z*) gene products, exhibited an opposite Dps-dependence (Table 4). Thus, the scale and type of Dps dependence are not universal for all tRNA fragments.

### 3.3. Fragments of Homologous Leucine and Serine tRNAs Exhibited Different Patterns of 3′-End Posttranscriptional Modifications

**Figure 3:**
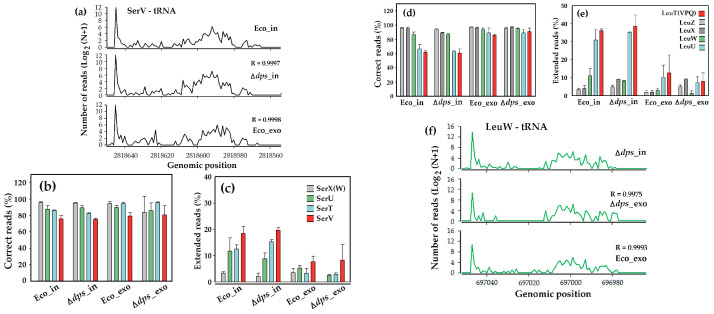
While being similarly fragmented (**a**,**f**), oligonucleotides derived from homologous tRNAs substantially differ at least in the efficiency of posttranscriptional extension of 3′-ends (**b**–**e**). (**a**,**f**) Normalized and averaged profiles representing the distribution of 5′-ends of fragmented tRNAs SerV and LeuW in different datasets. (**b**,**d**) Correct reads ascribed to the specified genes using Matcher software [50,55]. To estimate their rate among all reads derived from the target genes, “erroneous” oligonucleotides were collected using gene-specific 12-mer anchors, as illustrated in Figure 1a. (**c**,**e**) Reads were considered “extended” if they contained non-template incorporations or chimeras at the 3′-ends. Bars represent their normalized and averaged across similar datasets percentages for each tRNA type except reads from SerX(W) tRNAs in the Δ*dps*_exo dataset, which was too small in number for evaluation. R-values indicated in (**a**) and (**f**) reflect correlation with Eco_in and Δ*dps*_in samples, respectively.

To identify the structural or sequence features distinguishing homologous tRNAs with different dependence from Dps, we analyzed their fragmentation profiles, using the 5′-ends of precisely mapped reads as markers of endonucleolytic cleavage sites. We focused on serine and leucine tRNAs, as only SerV among the four serine tRNA types exhibited Dps dependence (Supplementary Table S2), while among the five leucine tRNAs, only LeuW avoided significant suppression in the secretomes (Table 4). However, visualization of fragmentation profiles of all these tRNAs and calculation of Pearson’s correlation coefficients as a metric of similarity revealed no discernible differences between cognate tRNAs with various Dps dependence. Surprisingly, fragmentation profiles even showed a strong correlation across the different strains and sample types (transcriptomes vs. secretomes; Figure 3a,f). This observation suggests that Dps is unlikely to be directly involved in either RNA degradation or its protection from nucleolytic cleavage.

Analysis of unmapped oligonucleotides, on the contrary, was more informative. Focusing on 3′ non-templated extensions as the main source of sequence deviations, we observed that the fraction of “correct” SerV-tRNA fragments precisely matching the genome was the smallest compared to oligonucleotides derived from other serine tRNAs with weaker dependence on Dps (Figure 3b). At the same time, the percentage of reads with 3′-end non-template extensions was the highest in all types of experiments (Figure 3c). By explaining the dependence of the number of correct reads, this also drew our attention to 3′-terminal modifications as potential discriminatory features. The situation with leucine oligonucleotides turned out to be even more interesting. Fragments derived from LeuZ and LeuX tRNAs exhibit the same pattern of distribution among correct and terminally extended reads as fragments of SerV tRNA (Figure 3d,e). Their dependence on Dps may be driven by an elevated proportion of erroneous sequences in the mutant strain’s secretome compared to the wild-type cells, while LeuW oligonucleotides may not meet the selection criteria due to the minimal error rate in the Δ*dps*_exo dataset (Figure 3e). Exceptionally low levels of correct reads among oligonucleotides derived from LeuU and identical LeuT, V, P, and Q tRNAs (further LeuTVPQ) in combination with unusually frequent non-template incorporations at their 3′-ends in transcriptomes (Figure 3d,e) represented a specific pattern of their distribution and prompted our focused investigations of LeuTVPQ fragments.

### 3.4. Fragments of Leucine tRNAs Exhibited Three Different Modes of 3′-End Modifications

**Figure 4:**
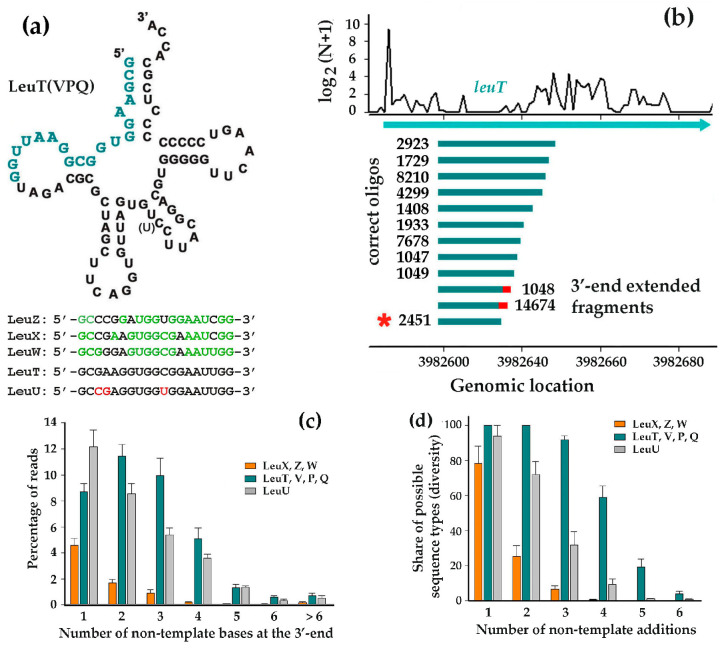
Being highly similar in sequence, the fragments of LeuTVPQ tRNAs vary in their posttranscriptional modifications. (**a**) Secondary structure of LeuT, V, P, and Q tRNAs with alignment of their identical 5′-terminal 19-mer compared to other leucine tRNAs fragments. Nucleotides matching LeuTVPQ are highlighted in green (LeuZ, X, W), while differing nucleotides in LeuU are marked in red. The single-nucleotide difference in the LeuP variable loop is indicated in brackets. (**b**) Top: 5′-ends distribution of 16–50 nt fragments derived from LeuT. Bottom: Size and abundance distribution of the most prevalent oligonucleotides (sample Eco_in_2), with red caps marking 3′-end non-template extensions in 19-mer marked by asterisk. (**c**) Mean percentage of reads with non-template nucleotides at the 3′-ends. Fragments of different tRNAs were collected individually using their common anchor sequence. Values for separately analyzed LeuU and LeuTVPQ tRNAs represent means across eight RNA-seq experiments. Values of LeuW, X, and Z were additionally averaged to show their common difference from the former two samples. (**d**) Diversity of 3′-end non-template motifs. For each library, motif richness was calculated as the percentage of observed unique sequences relative to all possible motifs with a particular length (4, 16, 64, and 256 for mono-, di-, tri, and tetranucleotides, respectively). Then, the values were averaged as described in (**c**).

In *E. coli*, leucine is encoded by five codons (CTA, CTC, CTG, TTA, and TTG) and is delivered to ribosomes by tRNAs transcribed from eight genes [57]. Four of them (leuTVPQ) share nearly identical sequences, all with anticodon CAG, differing only by a single nucleotide in the variable loop of LeuP (Figure 4a). 

They are organized into two operons: argX-hisR-leuT-proM and leuQ-leuP-leuV. Genes for LeuW (UAG) and LeuZ (UAA) are located within the operons metT-leuW-glnUW-metU-glnVX and glyW-cysT-leuZ-3′ETS, respectively. LeuX (CAA) is transcribed individually, while LeuU (GAG) is part of a unique *E. coli* operon (secG-leuU) that encodes both a protein and a tRNA. All leucine tRNAs undergo D-arm loop cleavage by endonucleases, but deviation analysis of their 5′-terminal fragments revealed distinct patterns of posttranscriptional modifications at the 3′-ends. The fragments from LeuX, LeuZ, and LeuW tRNAs displayed essentially the same size and copy number distributions of their 3′-end extensions in most of the analyzed samples (Supplementary Table S3). Typically, only one or two non-template nucleotides were detected in ~5% of reads, with longer extensions being progressively rarer. Thus, the most abundant oligonucleotides in the RNA-seq datasets are correct (Figure 3b,d).

In contrast, LeuTVPQ and LeuU fragments exhibited a significantly higher rate of reads with non-template motifs at their 3′-ends (Figure 4c). For LeuTVPQ, this primarily resulted from the consistent incorporation of random nucleotides to the growing end of the GCGAAGGTGGCGGAATTGG 19-mer (marked by an asterisk in Figure 4b). A similar phenomenon was at least partly observed for LeuU fragments from the highly similar 5′-terminal 19-mer GCCGAGGTGGTGGAATTGG (Figure 4a). Consequently, while LeuXZW fragments rarely contain even a single non-template nucleotide, LeuTVPQ fragments incorporated nearly all 64 possible triplets in one experiment and more than half of the 256 possible tetraplets (Figure 4d and Supplementary Table S3).

Most tetraplets found at the ends of other reads displayed predominant sequence motifs, leading us to classify them as “chimeras” resulting from illegitimate ligation of short oligos. However, LeuTVPQ-associated “chimeras” exhibited two distinct properties: (1) they were predominantly linked to the 3′-ends of specific 19-mers, and (2) in some cases, their stepwise elongation could be traced up to 26-mers. These observations suggest the potential involvement of a specific RNA polymerase in this process. While LeuU tRNA fragments also contained 3′-end extensions on their 5′-terminal 19-mer, these tails showed less diversity compared to LeuTVPQ reads (Figure 4d and Supplementary Table S3), implying some difference in the biogenesis mechanism for these two types of modifications.

### 3.5. LeuTVPQ tRNA Fragments Show Exceptional 3’-End Extension Diversity but Are Not Exclusive Substrates for This Modification

**Figure 5:**
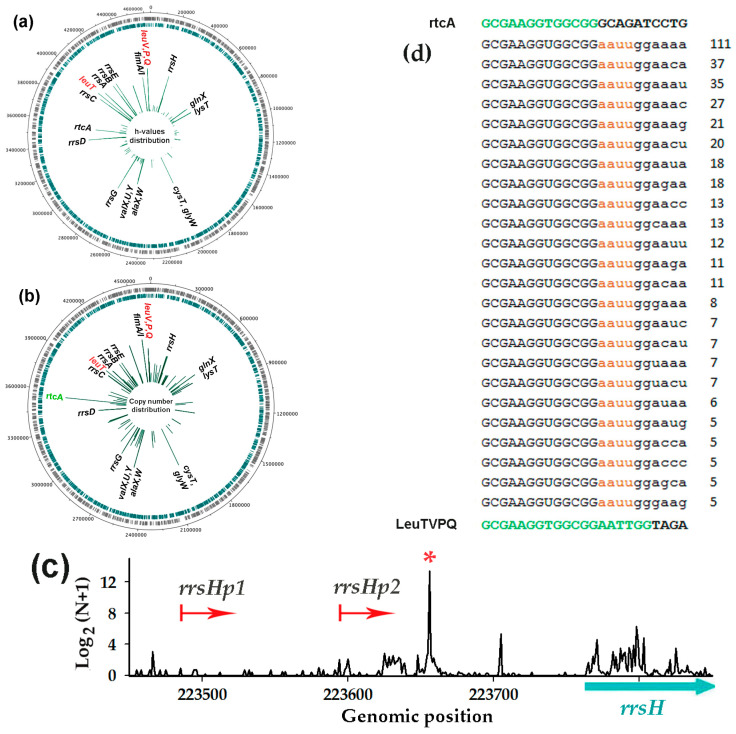
Although LeuTVPQ oligonucleotides exhibit the highest diversity, they are not the only substrates for terminal extensions (**a**–**c**) and contribute to the abundance of RNA products ascribed to the *rtcA* gene (**d**). (**a**,**b**) Profiles of the genomic loci, producing variable oligos (H-index > 2.0), plotted either by maximal H-values (**a**) or normalized copy numbers averaged across four cellular transcriptomes (**b**). (**c**) Normalized distribution of RNA-seq reads matching the genomic region near the *rrsH* ribosomal operon. Red arrows indicate transcription start points, and an asterisk marks the peak, corresponding to diversified RNAs. A logarithmic scale was used in all profiles. (**d**) Alignment of 23 nt reads representing modified fragments potentially derived from RtcA mRNA and LeuTVPQ tRNA. Genomic sequences of these products are shown above and below the alignment. Extended *k*-mers are marked in green.

It is already well-known that some bacterial RNAs are polyadenylated. While poly(A) polymerases primarily incorporate adenines, they occasionally add other nucleotides, with cytosine (C) being the most common. However, in oligos derived from LeuTVPQ, we observed an extraordinary variability in the 3’-terminal extensions (Supplementary Table S3), which only moderately followed the expected preference for A and C. To assess the uniqueness of the phenomenon, we developed the RandExt algorithm to identify reads with heterogeneous sequences (Figure 1b) and provide copy numbers and Shannon diversity indices (H) in its output files. The analysis was focused on short oligonucleotides (*k*-mers) ranging from 13 to 22 nucleotides in length, containing non-template flanks of 4–10 nt. Since secretomes usually possessed a lower level of reads with variable 3′-ends compared to transcriptomes (Figure 3c,e), we evaluated the uniqueness of the phenomenon using only the transcriptomic data of the wild-type strain and Δ*dps* mutant (Figure 5a,b). While selecting reads with variable ends across all four datasets using H > 2.0 as a threshold, we identified only 121 genomic loci as their potential origins (Figure 5a,b and Supplementary Table S4).

The output data varied depending on parameter combinations used (k-mer length and extension size L), affecting both read counts assigned to a specific position and the resulting H-value assessed from a particular program run. Due to the non-linear dependence of H-values on the profile of detected reads, they cannot be meaningfully averaged. We therefore used the maximal H-index in each genomic position revealed by using 56 parameter combinations (a total of 224 program runs for four datasets) and identified LeuTVPQ fragments as the most variable oligonucleotides (H = 4.46) (Figure 5a). LeuU fragments showed a significantly lower diversity (H = 1.95), falling below the threshold. The next significantly varied oligonucleotides (H = 4.14) were processed from an intergenic region flanking 16S rRNA genes (Figure 5c). This region is transcribed from two promoters located 286 and 178 bp upstream of the ribosomal operon. The transcripts are cleaved by an endonuclease 115 nt from the 5′-end of 16S rRNA (red asterisk in Figure 5c), predominantly yielding 22 nt oligonucleotide, which appeared to undergo substantial sequence diversification.

Being highly consistent with the distribution of the most variable RNA fragments Figure 5a), the profile based on their normalized abundances (Figure 5b) differed in the relative amplitudes of peaks. The most pronounced discrepancy was observed for reads mapped to the beginning of RtcA mRNA, encoding RNA 3′-terminal phosphate cyclase. RtcA fragment variability was only detectable using specific parameter combinations: short *k*-mers (13–14 nt) and long flanking regions (L = 7–10 nt). The identity of RtcA anchor 13-mer with the 5′-terminal 13-mer of LeuTVPQ tRNA, in combination with the absence of an AAUU motif in RtcA fragments (Figure 5d), means that most leucine tRNA fragments with non-templated extensions contribute to the observed abundance of fragments assigned to RtcA (Figure 5b), while their variability is lower (Figure 5a, H = 3.9). Although similar cross-contribution may take place for other RNA fragments, their limited occurrence will not hinder similar verification. In any case, the confirmed diversity of LeuTVPQ 3′-termini prompted us to assess their potential functionality.

### 3.6. Presence of Modified LeuTVPQ Fragments in Transcriptomes and Their Influence on E. coli Growth in Monoculture Are Medium-Dependent

Extraordinary diversity observed at the termini of one of the leucine tRNA fragments represents a new phenomenon, the biological role of which is only speculative. At this stage, the key question is whether further investigation is advisable. Assuming that the most sensitive indicator of the functionality of a new agent is its temporal or spatial specificity, we studied 16 additional RNA-seq libraries (PRJNA687658, GSE221667). Among these, 10 datasets derived from secretomes showed nearly undetectable levels of extended leucine tRNA fragments. This is probably one of the most important results of our study, suggesting that their localization is tightly regulated, though precluding their statistical analysis. The remaining six datasets (Eco_in_LB_1/2, Eco_Pr_1/2, and Eco_Rh_1/2) contained intracellular RNAs from *E. coli* grown in Luria–Bertani broth, either as a monoculture or in resource-shared conditions with *Prevotella copri* or *Rhodospirillum rubrum* (Table 2). Those co-cultures were maintained in membrane-separated compartments within a common chamber [52].

**Figure 6:**
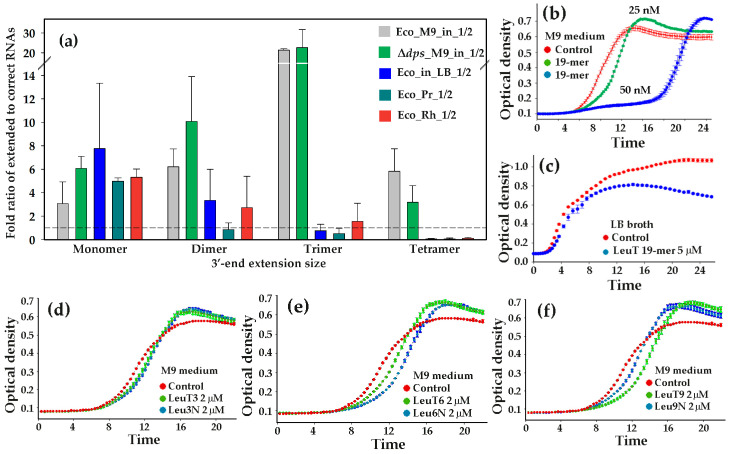
The culture medium influences the distribution of terminal extension lengths in LeuTVPQ fragments (**a**), which affects the functionality of the modified fragments (**d**–**f**). (**a**) Analysis of RNA-seq datasets targeted to evaluate the ratio of modified to unmodified RNA fragments. Six additional datasets analyzed in this part of the study are characterized in Table 2. Numbers of correct and modified LeuTVPQ 19-mers were individually estimated in each dataset and averaged across replicates. (**b**–**f**) Representative growth curves of *E. coli* in M9 (**b**,**d**–**f**) and Luria–Bertani media (**c**) in the presence and absence of model oligonucleotides.

The numbers of correct and modified LeuTVPQ 19-mers were estimated in 10 replicate experiments for oligonucleotides sized 20–23 nt, and their ratios were used as a metric reflecting excessive abundance of modified fragments (Figure 6a). We observed that modified 19-mers in cells grown in M9 medium (Eco_M9_in_1/2 and Δ*dps*_M9_in_1/2) always exceeded correct oligonucleotides in number. Among these, reads with non-templated trimers showed a 20-fold excess over correct 22 nt reads (Figure 6a). In contrast, transcriptomes from cells cultured in LB medium did not display such a pronounced accumulation of random 3 nt extensions in LeuTVPQ fragments. Although cells grown in LB had, on average, a sevenfold excess of oligonucleotides with incorrect terminal monomers, their ratio for longer extended RNAs fell below 1:1 even at the level of trimers (Figure 6a). Notably, the same distribution pattern of modified versus unmodified RNA fragments was observed in the transcriptomes [52] from *E. coli* cells grown adjacent to *P. copri* or *R. rubrum* (dark cyan and red bars). Thus, it is likely that the biogenesis of extended RNAs in cells depends on some specific components of LB medium that we have not been able to identify yet.

Figure 6b illustrates the impact of LeuTVPQ 19-mer on the *E. coli* growth dynamics in M9 medium. A concentration of mere 25 nM induced notable growth suppression, while 50 nM produced a clear bacteriostatic effect and promoted a higher cell density at the stationary phase compared to the control (Figure 6b). At the same time, significantly higher concentrations of synthetic oligonucleotides were required to inhibit *E. coli* growth in LB broth (Figure 6c). This inhibition proceeded without lag-phase extension and never exceeded the control cell density at the stationary phase. A similar dependence on the medium has already been reported for ciprofloxacin, which suppressed the growth of *E. coli* by 2–3 orders of magnitude less effectively in LB medium compared to M9 medium [58].

Longer oligonucleotides (LeuT3, LeuT6, and LeuT9), prepared as control samples for randomly extended synthetic Leu3N, Leu6N, and Leu9N, caused weaker growth delays compared to the 19-mer (Figure 6b,d–f), and random terminal trimers typically showed minimal or no effect on growth (Figure 6d). However, 6 and 9 nt extensions consistently altered *E. coli* growth dynamics in monocultures, retaining a typical M9 medium growth profile. Therefore, at the final stage of this study, we tested the influence of potentially functional Leu6N and Leu9N (Figure 6e,f) on the taxonomic composition of microbiomes transferred from laboratory rat feces to GMM.

### 3.7. 5′-Terminal 19-mers from LeuTVPQ Are Involved in Artificial Microbiome Shaping Depending on the Presence and Length of Their Random Extensions

**Figure 7:**
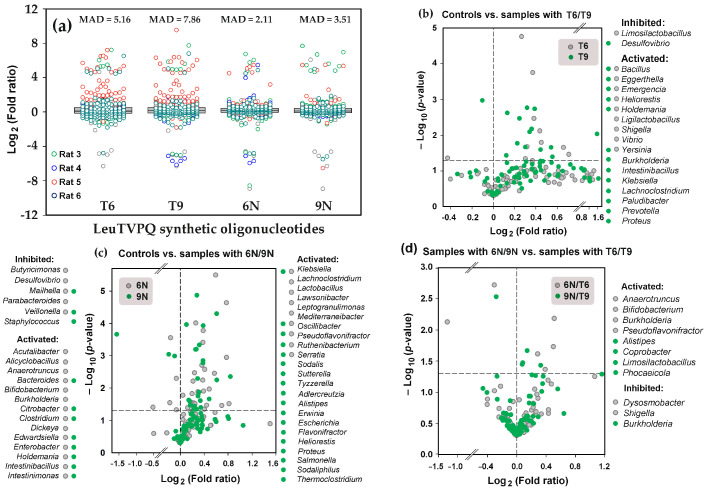
The 5′-terminal 19-mers of LeuTVPQ tRNAs extended by genomic nucleotides (T6, T9) or random sequences (6N, 9N) alter the taxonomic balance of the bacterial community revived from rat feces. (**a**) Box plots show fold change ratios (FCR) of individual taxa with ≥0.01 abundance, while mean absolute deviation (MAD) reflects their variance across all biological samples. (**b**–**d**) Volcano plots were generated based on at least four FCRs. Gray and green symbols represent the bacterial community response to oligonucleotides with 6 nt and 9 nt extensions, respectively. Horizontal dashed lines indicate a significance level of *p* = 0.05.

Assuming that random sequences in model nucleotides N6 and N9 might promote or enhance RNA interference in bacterial consortia, we added *R. rubrum* bacteria as an external species to assess their sensitivity to model oligos. This bacterium was introduced to the culture medium prior to inoculation to a final concentration of 4 × 10^6^ CFU/mL and was detected in all control samples with consistent persistence (0.0071 ± 0.0007). Following oligonucleotide supplementation, their relative abundance increased in all microbiomes, ranging from on average 1.49 ± 0.43-fold in three microbiomes to over 12-fold in some experimental consortia from rats 3 and 5 without apparent dependence on the type of 3′-end extensions. This response precluded *R. rubrum* application as a neutral reference taxon but likely reflects its specific reaction to oligonucleotide exposure. Nevertheless, the bacterial response to the oligonucleotides was sufficiently congruent to follow the adaptive reaction of nearly 100 genera, and many taxa in the artificial microbiome of rat 5 exhibited an increased abundance in response to oligos T6 and T9 compared with unexposed control samples (red symbols in Figure 7a). Their response to the randomly modified 6N and 9N appeared to be more consistent with bacteria of other samples. Decreased mean absolute deviation (MAD) values in the combined set of fold change ratios provided a quantitatively estimated consistency (Figure 7a).

While the introduction of oligonucleotides with the genomic sequence (T6 or T9) increased the abundance of many taxa, only 16 genera exhibited statistically significant expansion (Figure 7b). However, in most cases, the large increase (up to threefold, Log_2_ = 1.58) was statistically insignificant. Minor suppression, in contrast, was registered only for *Limosilactobacillus* and *Desulfovibrio*. It should be considered, however, that dead cells also contribute to the rate of detected bacteria. Thus, the number of inhibited taxa is underestimated.

Terminally diversified LeuTVPQ oligos decreased variability in the bacterial responses (Figure 7a), thereby increasing the number of both reliably activated and suppressed taxa (Figure 7c). Although only 5 of 16 genera from panel (b) appeared in the set of bacteria stimulated by diversified oligonucleotides (panel c), the other eight of them were also activated in the microbiomes of rats 4 and 5. Thus, the data were rather consistent. We then compared the effects of oligonucleotides with random 3′-end extensions and those having genomic sequences at the end (Figure 7d). Presumably identifying taxa with direct dependence on random extensions, we observed eight genera with a higher abundance compared to samples supplemented with correct oligonucleotides. Five of them exhibited the same difference with blank controls as in Figure 7c (*Anaerotruncus*, *Bifidobacterium*, *Burkholderia*, *Pseudoflavonifractor*, and *Alistipes*), indicating a stimulatory effect of both the oligonucleotide per se and its random extensions. In *E. coli* monoculture, such a strengthened unidirectional effect was demonstrated by 9N compared to T9 (blue and green curves in Figure 6e, respectively). On the contrary, the stimulatory effect of T6 for *Shigella* (Figure 7b) was attenuated by 6N (Figure 7d). In the monoculture of *E. coli*, this situation was reproducibly realized for T9 and 9N (blue and green curves in Figure 6f, respectively). For *Burkholderia*, with T9-activated growth compared to untreated microbiomes (Figure 6b), 9N was a suppressor, while 6N had a stimulatory influence (Figure 6d). Therefore, not only 5′-terminal fragments of LeuTVPQ tRNAs but also their 3′-end extensions may play a regulatory role in bacterial communities.

## 4. Discussion

In eukaryotes, 3′-terminal mRNA elongation by predominantly adenine residues (polyadenylation) is a key and well-studied process of mRNA maturation. In addition to poly(A) polymerase (PAP), efficient polyadenylation requires the participation of several other proteins. These include the cleavage and polyadenylation specificity factor (CPSF), which recognizes an A/U-rich signal located 10–30 nt upstream of the polyadenylation site in pre-mRNA, and the cleavage stimulatory factor (CstF), which binds a U/GU-rich downstream sequence element (DSE) located approximately 40 nt away [59]. Adenine incorporation is tightly coupled with endonucleolytic cleavage of premature mRNAs [60], and the process occurs in two phases. During the first phase, PAP alone slowly incorporates a limited number of nucleotides, dissociating from the RNA after each polymerization step. Rapid elongation is then triggered when CPSF and the nuclear poly(A)-binding protein (PABPN1) bind to this initial oligo(A) tract. This binding recruits additional factors to form an active processing complex [61] that synthesizes a standard-length poly(A) tail of approximately 250 nucleotides [62] and protects mRNAs from degradation.

Although polyadenylation in bacteria is much less investigated, it is already acknowledged that the majority of *E. coli* mRNAs are also to some extent polyadenylated [63]. Microarray analysis, for instance, revealed 90% of *E. coli* mRNAs, which undergo some degree of polyadenylation by poly(A) polymerases I (PAP I) either as full-length transcripts or decay intermediates [27]. It is generally accepted that microbial poly(A) polymerases can initiate polymerization simply by recognizing a free 3′-OH group of RNA substrates, without strict sequence or structural requirements [24]. However, analysis of over 240 transcripts suggested Rho-independent transcription terminators as potential polyadenylation signals for PAP I [27]. In *E. coli*, PAP I adds up to 50 adenines to the 3′-termini of RNAs [29], which typically provokes decay via recruiting exoribonucleases such as RNase II and PNPase but can also stabilize mRNAs by competitive elongation of their 3′-termini. In terms of our study, it is important that in vivo PAP I-synthesized tails exclusively contain A residues [27]. Thus, PAP I is unlikely to be considered as a potential candidate for the role of the enzyme diversifying LeuTVPQ.

Two other *E. coli* enzymes, polynucleotide phosphorylase (PNPase) and RNAse PH, can either degrade RNAs using inorganic phosphate (Pi) or extend their 3′-ends using nucleotide diphosphates [64]. Both enzymes synthesize heterogeneous 3′-tails similar to those we discovered in LeuT(VPQ). However, this synthesis is highly dependent on intracellular Pi levels. The high Pi concentration in M9 medium should promote the degradative activity of these enzymes, while more than an order-of-magnitude-lower phosphate concentration in Luria–Bertani broth should favor polymerization [65]. Our RNA-seq data showed a strong inverse relationship to this model (Figure 6a), which likely excludes PNPase and RNAse PH from being responsible for the terminal diversification we observed. Since the functionality of the second poly(A) polymerase (PAP II) predicted in *E. coli* [66] has not yet been confirmed [28,63], and tRNA nucleotidyl transferase adding the CCA aminoacyl-binding triplet at the end of tRNAs incorporates only C and A [67], the enzyme responsible for diversifying LeuT(VPQ) fragments should probably be searched among proteins with unknown functions.

While analyzing oligonucleotides from several dozen RNAs across eight RNA-seq datasets, we identified three patterns of terminal extensions. All of them are represented by the eight leucine tRNA fragments exemplified in Supplementary Table S3. Oligonucleotides derived from LeuZ and LeuX represent typical extension patterns. Their fragment sets contained only 2.97% and 6.02% of oligonucleotides with non-template additions, respectively. Most of these additions have no more than one incorrect nucleotide in the terminal trinucleotide. While several longer extensions (L ≥ 3) were observed, most were present in only a single copy. Among the fragments from LeuX tRNA, not a single oligonucleotide met the criteria for random extension, requiring no substitutions in the terminal tetranucleotide and L-4 substitutions in longer extensions. Therefore, none were identified by RandExt as randomly extended. Among reads from LeuZ, there were seven such oligonucleotides (marked by asterisks in Supplementary Table S3), but they all belonged to fragments of different length (L), hence their Shannon diversity indices (H) could not be estimated. Despite a substantially larger number of transcripts from LeuW, the percentage of extended oligonucleotides among its fragments was almost the same as for LeuX (6.84%), and there were very few reads of similar length with L ≥ 3. Thus, products of LeuW were also classified as undiversified. In the case of LeuU, the percentage of extended reads was notably higher (25.81%). This allowed for the estimation of the H-value (1.475) for seven 23-mers with variable tetramers at their ends. Although the maximal H-values observed across all experiments were 1.95, the fragments from LeuU also did not exceed the threshold level required to be classified as heterogeneous, but they differed from the first group because they provided reads for H-value estimation. The percentage of extended reads among LeuTVPQ products (35.2%) was only slightly higher than that of LeuU fragments (25.81%). However, for their 5’-terminal 19-mers, the number of extended fragments was nearly six times greater than the number of unmodified oligonucleotides.

Based on this classification, we obtained 102 oligonucleotides, with diversity higher than for LeuU (Supplementary Table S4). Excluding the product of *rtcA*, 8 of 22 RNAs with highly heterogeneous 3’-ends (H > 3.0) originated from tRNAs, while the remaining 14 were processed from polycistronic transcription units of ribosomal operons and from their leader sequences (Supplementary Table S4). Complex endonuclease cleavage is part of their maturation process [68], which is obligatory for eukaryotic poly(A) polymerases but is not strictly required for the bacterial PAP activity. Thus, it is likely that their terminal diversification is directly coupled with processing. However, the question of whether random extensions are signals for degradation or not remains open. Since randomly modified LeuTVPQ fragments were significantly enriched in the bacterial transcriptomes, compared to their unmodified counterparts, yet were nearly absent from the secretomes, their targeted diversification for intracellular use appears to be worthwhile. In cells, oligonucleotides with variable termini could bind diverse cellular RNAs, potentially facilitating elimination of mRNAs uninvolved in translation through an RNA interference-like mechanism. On the other hand, forced diversification inevitably generates sequences absent in the *E. coli* genome. Such oligonucleotides can constitute a primitive defense system: while lacking adaptive memory, they could provide alertness against foreign nucleic acids when even imperfect base pairing with invader molecules might become the frontline to delay the infection.

By identifying RNA fragments exhibiting Dps-dependent secretion, we provide evidence supporting its potential role in RNA export. This finding expands the functional repertoire of Dps as a “moonlighting” protein performing diverse functions using a single structural domain [69]. Thus, being a major nucleoid protein during steady-state growth [70], Dps also acts as a ferritin, oxidizing toxic ferrous ions and sequestering Fe(III) within an internal cavity formed by 12 identical subunits [71]. Furthermore, during the stationary phase, Dps mediates bacterial nucleoid condensation through the involvement of positively charged amino acid residues in its 12 unstructured N-terminal modules [72]. Electrostatically attaching to the negatively charged DNA backbone, Dps can interact with any genomic locus. However, ChIP-seq [44] and in vitro SELEX [73] experiments revealed a non-random distribution pattern of Dps binding sites throughout the bacterial chromosome. Based on these data, supported by atomic force microscopy [46], regions capable of forming hairpin structures have been suggested as preferred Dps binding sites. This structure-specific interaction could explain the dependence of the genome expression profile on the presence of Dps (Figure 2b). However, while Dps condenses the genome into compact multilayered structures in starving cells, its overproduction from a recombinant gene does not cause the same packaging during exponential growth. This discrepancy may be attributed to several factors: the presence of nucleoid protein Fis, which is replaced by Dps in the stationary phase [74], a high concentration of Mg^2+^, which prevents heterochromatization during rapid growth [37] and interference from RNA [40].

The ability of Dps to interact with RNA was first reported by Wolf et al. in 1999 [36]. Later, Bykov et al. [39] demonstrated that purified Dps immobilized on acrylate beads could bind short fragments of transfer and small regulatory RNAs in vitro. Therefore, to establish the role of Dps in delivering short RNAs outside the cell, the remaining key question is whether it can cross the cell membrane. For *E. coli* Dps, this is currently supported only by indirect evidence. For instance, exposure of *E. coli* MG1655 to bacteriophages resulted in the selection of phage-tolerant bacteria with an increased propensity for biofilm formation, which accumulated Dps in the outer membrane fraction [41]. Another evidence comes from a study of Li et al. [75], who analyzed outer membrane proteins involved in streptomycin resistance in *E. coli* and identified Dps as a protein with reduced abundance in the membrane of an antibiotic-resistant strain. The identification of 102 oligonucleotides derived from structured RNAs and exhibiting Dps-dependent secretion (Supplementary Table S2) is consistent with all previously obtained data and provides new evidence of Dps participation in RNA export. Given the ability of Dps to bind both DNA and RNA, we propose that Dps may capture short RNAs immediately after their synthesis and facilitate their export prior to terminal diversification. This model explains both the significantly reduced level of terminally modified oligonucleotides in the secretomes compared to the transcriptomes (Figure 3b,c) and the dramatic decline in correlation between the wild-type and Δ*dps* exoRNAs profiles (Figure 2e).

The most unexpected finding of this study is the clear dependence of model oligonucleotides’ impacts on growth medium composition. Similar effects have been observed for bactericidal ciprofloxacin and bacteriostatic chloramphenicol [58], of which chloramphenicol inhibited bacterial metabolism in both M9 medium and LB broth, whereas the addition of ciprofloxacin allowed *E. coli* cells cultured in LB medium to retain metabolic activity at significantly higher concentrations than those in M9 medium. The toxicity of ciprofloxacin is mediated by its ability to stabilize the gyrase-DNA cleaved complex, thereby preventing DNA supercoiling and relaxation [76], which is not immediately lethal but induces the SOS response and suppresses replication and transcription. Chloramphenicol, on the contrary, inhibits protein synthesis [77], resulting in rapid growth suppression through multiple biochemical alterations. The data obtained by Smirnova et al. [58] indicated that the primary difference in the physiological response of *E. coli* to bacteriostatic and bactericidal antibiotics is the rate at which the metabolic processes are inhibited. The dependence of LeuTVPQ action on the culture medium was similar to ciprofloxacin, which is reasonable to expect from oligonucleotides without obvious toxicity. Given the potential toxicity of diversified LeuTVPQ fragments, it is significant that *E. coli* cells accumulate RNA fragments with heterogeneous ends specifically under conditions when sensitivity to their bacteriostatic effect is heightened. 

Synthetic analogs of LeuTVPQ oligonucleotides consistently caused reproducible effects in *E. coli* monocultures cultivated in vivo and revealed statistically significant alterations in several dozen taxa from microbiota consortia transferred from rat fecal material to GMM. Notably, among the limited number of taxa responding to 3′-randomized oligonucleotides, we observed a selective activation of potentially probiotic *Bifidobacteria* and inhibition of potentially pathogenic *Shigella* species, denoting possible therapeutic applications. In monoculture experiments, *E. coli* exposed to synthetic oligonucleotides suffered growth suppression during logarithmic phase but outcompeted the control samples at the stationary phase without essential changes in the total culture density. Consequently, in ex vivo experiments, *E. coli* also maintained stable population levels, changing on average by 1.26 ± 0.12 times from 3.99 ± 0.67% in the control samples. This consistency paves the way for the implementation of revived microbiomes for an extensive search of taxa responding to novel agents of biotechnological or medical significance.

## 5. Conclusions

We undertook the presented study in an attempt to reveal and systematically characterize posttranscriptional modifications in *E. coli* oligonucleotides with a potential regulatory or signaling role. Discovering unusual diversification in 5′-terminal fragments of LeuTVPQ, we made a few steps towards understanding this phenomenon, demonstrating the uniqueness of LeuTVPQ fragments and evaluating the functional relevance of their modifications. While the biological role of random extensions remains to be fully elucidated, our findings provide a foundation for future studies to unravel the mechanistic and physiological significance of modifications evolutionary attuned to increase biodiversity.

## Figures and Tables

**Table 1:** Sequencing statistics of RNA samples obtained from the transcriptomes and secretomes of the wild-type strain *E. coli* K-12 MG1655 and its Δ*dps* mutant (Project NCBI GEO GSE221667).

Sample	Number of Reads			Description	Data Availability
	Before QC	After QC	16 ≤ L ≤ 50		
Eco_in_M9_1 *	1,546,157	996,435	821,483	RNAs isolated from *E. coli* MG1655 cells grown in M9 medium to OD = 0.4–0.6	GSM6892281 GSM6892282
Eco_in_M9_2	2,494,971	1,528,878	1,517,245		
*dps*_del_in_1 *	2,093,000	1,294,678	1,292,272	RNAs isolated from *E. coli* Δ*dps* mutant cells grown in M9 medium to OD = 0.4–0.6	GSM6892285 GSM6892286
*dps*_del_in_2	1,771,628	1,082,635	1,072,005		
Eco_exo_1 *	1,832,829	890,000	788,543	RNAs isolated from M9 medium used for culturing *E. coli* MG1655 cells	GSM6892283 GSM6892284
Eco_exo_2	1,220,822	737,402	729,656		
*dps*_del_exo_1 *	608,759	182,984	158,810	RNAs isolated from M9 medium used for culturing *E. coli* Δ*dps* mutant cells	GSM6892287 GSM6892288
*dps*_del_exo_2	917,604	258,455	180,502		

* RNA-seq experiments performed simultaneously.

**Table 2:** RNA-seq datasets used to assess the dependence of the terminal processing on growth conditions.

Sample	Number of Reads		Growth Conditions	BioProject PRJNA687658 Sample Description and References
	Before QC	After QC		
Eco_in_LB_1 Eco_in_LB_2	1,320,485 2,305,864	299,167 1,589,066	Anaerobic growth in Hungate tubes	RNAs from *E. coli* MG1655 cells grown individually in LB medium supplemented with L-cysteine HCl after inoculation with an overnight culture at a 1:4000 dilution. Cells were harvested at OD_600_ = 0.4 [33].
Eco_Pr_1 Eco_Pr_2	2,629,571 864,537	1,563,678 652,497	Anaerobic growth in membrane-separated chamber	RNAs of *E. coli* MG1655 cells grown in separate compartments with *Prevotella copri* or *Rhodospirillum rubrum* in LB medium + L-cysteine HCl. *P. copri* and *R. rubrum* were inoculated at a 1:10 ratio with 48 and 24 h cultures, respectively. *E. coli* cells were harvested at OD_600_ = 0.4 [52].
Eco_Rh_1 Eco_Rh_2	1,301,404 1,176,395	1,028,350 681,123		

**Table 3:** Sequences of synthetic analogs used for functional analysis.

Name	5′-Terminal Sequences of the Fragments of Four Leucine tRNAs
LeuTVPQ	5′-GCGAAGGUGGCGGAAUUGG-3′
LeuTVPQ_T3	5′-GCGAAGGUGGCGGAAUUGGUAG-3′
LeuTVPQ_3N *	5′-GCGAAGGUGGCGGAAUUGGNNN-3′
LeuTVPQ_T6	5′-GCGAAGGUGGCGGAAUUGGUAGACG-3′
LeuTVPQ_6N	5′-GCGAAGGUGGCGGAAUUGGNNNNNN-3′
LeuTVPQ_T9	5′-GCGAAGGUGGCGGAAUUGGUAGACGCGC-3′
LeuTVPQ_9N	5′-GCGAAGGUGGCGGAAUUGGNNNNNNNNN-3′

* nucleotides randomly incorporated in RNA in the presence of all 4 substrates.

**Table 4:** Genomic regions encoding tRNAs with and without Dps-dependent secretion.

Peak Maximum	Strand	Number of Reads *				Genomic Loci			Strand
		Transcriptome		Secretome		Gene(s)	Borders		
		w.t.	Δ*dps*	w.t.	Δ*dps*		Right	Left	
225,381	+	941	638	2318	966	*ileV*	225,381	225,457	+
3,427,152	-	618	718	1204	1109	*ileU*	3,427,076	3,427,152	-
4,037,141	+	618	718	1159	966	*ileT*	4,037,141	4,037,217	+
564,723	+	137	113	477	169	*argU*	564,723	564,799	+
2,817,860	-	422	386	1311	1662	*argQ*(*ZYV*)	2,817,784	2,817,860	-
3,423,655	-	1387	2127	4380	4053	*thrV*	3,423,580	3,423,655	-
696,740	-	1499	2822	2310	2490	*metU*(*T*)	696,664	696,740	-
2,947,387	+	408	307	1182	501	*metZ*(*WV*)	2,947,387	2,947,463	+
3,318,289	-	413	316	1265	616	*metY*	3,318,213	3,318,289	-
697,047	-	12,294	14,261	1730	1652	*leuW*	696,963	697,047	-
780,765	+	1046	941	473	357	*valT*(Z)	780,765	780,840	+
1,031,712	-	1354	2065	1233	1076	*serT*	1,031,625	1,031,712	-
1,097,652	-	2279	3837	818	724	*serX*(*W*)	1,097,565	1,097,652	-
2,043,557	-	432	624	198	148	*serU*	2,043,468	2,043,557	-
2,521,253	+	1868	2918	1576	1603	*lysV*	2,521,253	2,521,328	+
2,729,444	-	286	398	460	391	*gltW*	2,729,369	2,729,444	-

* Deviations from the required parameters are color-highlighted depending on their strength.

## Data Availability

Previously obtained RNA-seq data are available at the SRA database of NCBI (NCBI GEO Project and GSE221667). Amply-Seq libraries for taxonomic analysis generated in this study are available at the SRA database of NCBI (BioProject PRJNA1291452).

## Supplementary Materials

The following supporting information can be downloaded at https://www.mdpi.com/article/10.3390/microorganisms13092189/s1. Table S1: Sequencing statistics of Amply-Seq carried out for ex vivo cultured bacteria; Table S2: Genomic loci producing oligonucleotides with Dpa-dependent secretion; Table S3: 3′-Terminal diversity of reads related to leucine tRNAs with extended 3′-ends; Table S4: RNA fragments identified in the *E. coli* transcriptomes with 3′-terminal non-template extensions.
