# Supplementary material for: Posttranscriptional 3′-Terminal Modifications of Escherichia coli RNA Fragments Evolved for Diversity Boosting
Source: Microorganisms. 2025 Sep 19;13(9):2189. doi: 10.3390/microorganisms13092189 (PMC12472396; doi:10.3390/microorganisms13092189)
Supplement: Supplementary file 1 [file microorganisms-13-02189-s001.zip › Supplementary Table S1.pdf]

Supplementary Table S1 Sequencing statistics of Amply-Seq carried out for ex-vivo cultured bacteria

| Sample | Number of amplicons (reads) | |
|---|---|---|
| | Total | Classified |
| **Rat № 1** | | |
| R1-contr | 260,875 | 245,564 |
| R1-T6 | 217,996 | 203,684 |
| R1-T6N | 234,839 | 220,860 |
| R1-T9 | 239,373 | 226,392 |
| R1-T9N | 240,525 | 225,283 |
| **Rat № 3** | | |
| R3-contr | 259,072 | 246,285 |
| R3-T6 | 435,823 | 414,638 |
| R3-T6N | 235,582 | 223,836 |
| R3-T9 | 269,193 | 253258 |
| R3-T9N | 363,942 | 342733 |
| **Rat № 4** | | |
| R4-contr | 397,825 | 372,382 |
| R4-T6 | 373,578 | 350,766 |
| R4-T6N | 265,196 | 246,249 |
| R4-T9 | 265,948 | 245,171 |
| R4-T9N | 329,129 | 305,766 |
| **Rat № 5** | | |
| R5-contr | 243,816 | 223,751 |
| R5-T6 | 421,788 | 385,724 |
| R5-T6N | 345,090 | 313,489 |
| R5-T9 | 276,606 | 251,788 |
| R5-T9N | 245,504 | 224,426 |
| **Rat № 6** | | |
| R6-contr | 254,318 | 215,231 |
| R6-T6 | 284,511 | 248,823 |
| R6-T6N | 295,065 | 251,157 |
| R6-T9 | 336,471 | 292,389 |
| R6-T9N | 384,502 | 327,518 |
